# Analysis of the Actions of RARγ Agonists on Growing Osteochondromas in a Mouse Model

**DOI:** 10.3390/ijms25147610

**Published:** 2024-07-11

**Authors:** Sonia A. Garcia, Kimberly Wilson, Ningfeng Tang, Hongying Tian, Takeshi Oichi, Aruni T. Gunawardena, Michael Chorny, Ivan S. Alferiev, John E. Herzenberg, Vincent Y. Ng, Masahiro Iwamoto, Motomi Enomoto-Iwamoto

**Affiliations:** 1 Department of Orthopaedics, School of Medicine, University of Maryland, Baltimore, MD 21201, USA; soniagarcia0119@gmail.com (S.A.G.); kimberly.wilson@som.umaryland.edu (K.W.); ntang@som.umaryland.edu (N.T.); htian@som.umaryland.edu (H.T.); oichi-tky@umin.ac.jp (T.O.); vng@som.umaryland.edu (V.Y.N.); masahiro.iwamoto@som.umaryland.edu (M.I.); 2 Department of Orthopedics, Teikyo University School of Medicine, Tokyo 1738608, Japan; 3 Department of Biomechanics, Northeast College of Health Sciences, Seneca Falls, NY 13148, USA; aruni.tiffany@gmail.com; 4 Department of Pediatrics, The Children’s Hospital of Philadelphia, Philadelphia, PA 19104, USA; chorny@chop.edu (M.C.); alferiev@chop.edu (I.S.A.); 5 International Center for Limb Lengthening, Rubin Institute for Advanced Orthopedics, Sinai Hospital of Baltimore, Baltimore, MD 21215, USA; jherzenb@lifebridgehealth.org

**Keywords:** osteochondroma, cartilage tumor, chondrocytes, palovarotene, retinoic acid nuclear receptor gamma

## Abstract

The actions of the retinoic acid nuclear receptor gamma (RARγ) agonist, palovarotene, on pre-existing osteochondromas were investigated using a mouse multiple osteochondroma model. This approach was based on the knowledge that patients often present to the clinic after realizing the existence of osteochondroma masses, and the findings from preclinical investigations are the effects of drugs on the initial formation of osteochondromas. Systemic administration of palovarotene, with increased doses (from 1.76 to 4.0 mg/kg) over time, fully inhibited tumor growth, keeping the tumor size (0.31 ± 0.049 mm^3^) similar to the initial size (0.27 ± 0.031 mm^3^, *p* = 0.66) while the control group tumor grew (1.03 ± 0.23 mm^3^, *p* = 0.023 to the drug-treated group). Nanoparticle (NP)-based local delivery of the RARγ agonist also inhibited the growth of osteochondromas at an early stage (Control: 0.52 ± 0.11 mm^3^; NP: 0.26 ± 0.10, *p* = 0.008). Transcriptome analysis revealed that the osteoarthritis pathway was activated in cultured chondrocytes treated with palovarotene (Z-score = 2.29), with the upregulation of matrix catabolic genes and the downregulation of matrix anabolic genes, consistent with the histology of palovarotene-treated osteochondromas. A reporter assay performed in cultured chondrocytes demonstrated that the Stat3 pathway, but not the Stat1/2 pathway, was stimulated by RARγ agonists. The activation of Stat3 by palovarotene was confirmed using immunoblotting and immunohistochemistry. These findings suggest that palovarotene treatment is effective against pre-existing osteochondromas and that the Stat3 pathway is involved in the antitumor actions of palovarotene.

## 1. Introduction

Osteochondroma is the most common benign bone tumor, accounting for 20–50% of benign bone tumors in children [1]. Patients may develop solitary osteochondromas due to injuries at the metaphysis or multiple osteochondromas associated with mutations in the *Exostosin 1* (*EXT1*) and *2* (*EXT2*) genes, a condition known as hereditary multiple osteochondromas (HMO; previously called hereditary multiple exostoses, HME) [2,3]. Osteochondromas are not present at birth in patients with HMO. However, approximately 96% of affected individuals develop multiple osteochondromas by 12 years of age [4].

In most cases, osteochondromas are diagnosed using radiography or computed tomography (CT) [1]. Patients with HMOs currently rely on surgery for treatment. Patients undergo an average of 2.7 surgeries in their lifetime for osteochondromas [5]. However, repetitive surgeries pose a severe burden on patients, families, and the healthcare system. Depending on the location of the osteochondroma, the surgical procedures can vary from simple to complex. If the entire cartilaginous cap is not removed, osteochondromas can recur or malignant transformation may occur [6]. Preventing surgery will minimize recovery time and healthcare costs and reduce the risk of complications.

To date, three drugs have been demonstrated to inhibit osteochondroma formation in mouse models of multiple osteochondroma (MO) models. They are LDN-193189, a bone morphogenetic protein antagonist [7], PF-04449913, an inhibitor of Indian hedgehog [8], and palovarotene, a specific agonist of retinoic acid nuclear receptor gamma (RARγ) [9]. However, no Food and Drug Administration (FDA)-approved drugs are currently available for the treatment of osteochondromas. A clinical phase 2 trial (NCT03442985) was performed to investigate the efficacy and safety of palovarotene for the treatment of MO. However, a clinical hold for this trial was issued following safety reports of early growth plate closure in pediatric patients observed during another clinical trial (NCT02190747) for a lethal genetic disease, fibrodisplasia ossificance progressiva (FOP) [10,11]. Although the FDA has approved palovarotene as a treatment for patients with FOP (NDA215559), studies on the clinical application of palovarotene for MO have remained limited.

This study was performed to examine the application of palovarotene and other RARγ agonists for the treatment of osteochondromas. The study has three aims. One is to determine whether palovarotene inhibits the growth of pre-existing osteochondromas because patients generally visit clinicians after osteochondroma formation is detected. The second aim is to determine whether local application of the RARγ agonist can inhibit osteochondroma growth, minimizing systemic adverse effects. The third aim is to investigate alterations of the molecular pathways in palovarotene-treated osteochondroma chondrocytes because elucidation of molecular pathways affected by the RARγ agonist should be valuable in developing alternative drugs for osteochondromas. We observed that palovarotene halted the growth of pre-existing osteochondromas in increasing doses with age. Furthermore, local application of the RARγ agonists inhibited osteochondroma growth for a limited period of time. In addition, we demonstrated that RARγ agonists stimulated the signal transducer and activator of the transcription 3 (Stat3) signaling pathway in osteochondroma cells.

## 2. Results

### 2.1. Measurement of Cartilage Tumor Volume by Enhanced microCT in an AcanCreER;Ext1f/f Mouse Osteochondroma Model

**Figure 1:**
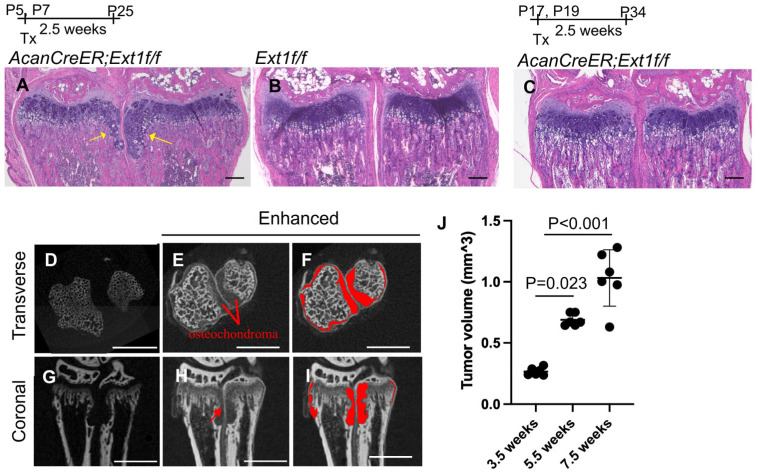
Induction of osteochondromas in the wrists of *AcanCreER;Ext1f/f* mice. *AggrecanCre^ERT2^* (*AcanCreER*) mice were crossed with *Ext1^e2neofl^* (*Ext1f/f*) to generate *AcanCreER;Ext1f/f* mice. The *AcanCreER;Ext1f/f* mice (**A**,**C**–**J**) and the littermate *Ext1f/f* mice (**B**) received tamoxifen injections at P5 and P7 (**A**,**B,D**–**J**) or P17 and P19 (**C**). Forelimbs were harvested at 3.5 weeks (**A**–**C**,**J**), 5.5 (**D**–**J**), or 7.5 weeks (**J**) after completion of tamoxifen injections. (**A**–**C**) Hematoxylin and eosin (HE) staining of coronal sections. Yellow arrows indicate osteochondromas. The bars denote 200 μm. (**D**–**I**) Transverse (**D**–**F**) and coronal views (**G**–**I**) of the microCT images of the forelimbs; untreated (**D**,**G**) and treated (**E**,**F**,**H**,**I**) with phosphotungstic acid. The red areas indicate osteochondromas (**F**,**I**). The bars denote 1 mm. (**J**), Volumes of osteochondromas in the distal epiphysis of the ulna and radius were measured and sum (*n* = 5, 3.5 weeks; *n* = 5, 5.5 weeks; *n* = 6, 7.5 weeks).

Various mouse osteochondroma models have been reported [12]. We chose the *AcanCreER;Ext1f/f* compound transgenic mouse model [7] generated by crossing *AcanCreER* mice with *Ext1^e2neofl^* mice. In this transgenic mouse line, tamoxifen-dependent Cre recombination can be induced in neonates and at older ages. To confirm the efficiency and selectivity of Cre recombination in *AcanCreER* mice, tamoxifen was injected into *AcanCreER;R26R^ZsGreen^* mice starting at the neonatal (P5) and infant (P17) stages. In both cases, Cre recombination in the growth plate was efficiently and selectively induced three days after tamoxifen injection, as evidenced by the expression of ZsGreen reporter proteins (Supplementary Figure S1A–D). When tamoxifen was injected at P5 and P7, ectopic cartilage masses, referred to as osteochondromas, were observed in the periphery of the proximal growth plate in the ulna and radius in *AcanCreER;Ext1f/f* mice (Figure 1A, yellow arrows) but not in *Ext1f/f* mice (Figure 1B) 2.5 weeks after the injections, indicating that tumor formation after tamoxifen injections is dependent on the *AcanCreER* transgene. However, the induction of ectopic cartilage was much less prominent when tamoxifen was injected into P17 (Figure 1C). Similar differences in the degree of osteochondroma induction were observed at the costochondral junctions of the ribs (Supplementary Figure S1E; yellow arrows vs. Supplementary Figure S1F). These results indicate that the effective induction of osteochondromas in the *AcanCreER;Extf/f* mouse model requires Cre recombination at the neonatal stage. Sex-based differences were observed in the degree of osteochondroma formation (Supplementary Figure S2A,C). In this study, we induced osteochondromas in *AcanCreER;Extf/f* male mice, starting at P5.

One pitfall in the analysis of osteochondromas is the difficulty of accurately determining their volume. Previous studies have used histomorphometric analysis and the number of ectopic cartilage masses to evaluate tumor formation [7,9,13]. In this study, we used a phosphotungstic acid (PTA)-enhanced microCT. Without PTA staining, microCT only recognized the bone, as shown in the transverse and coronal planes (Figure 1D and Figure 1G, respectively). After PTA staining, reconstructed microCT images showed both bone and cartilaginous osteochondromas located in the ulna and radius of the distal forelimb (Figure 1E,H; Figure 1F,I, marked in red). We could distinguish the perichondrium from the osteochondromas (Figure 1H, red arrow). The osteochondroma region was manually defined, and the entire volume of the selected region was determined. Increases in the volume of tumors from 3.5 weeks of age (2.5 weeks after tamoxifen injection) to 7.5 weeks of age (6.5 weeks after tamoxifen injection) were evident (Figure 1J and Supplementary Table S1).

### 2.2. Inhibition of Pre-Existing Osteochondromas by Palovarotene

**Figure 2:**
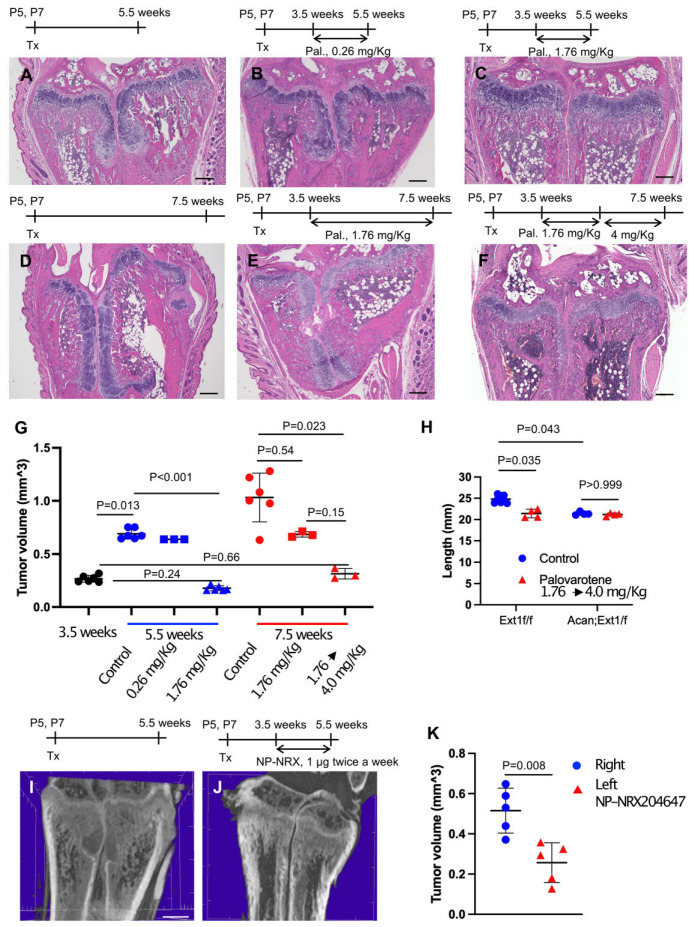
Administration of palovarotene inhibits osteochondroma growth. *AcanCreER;Ext1f/f* mice (**A**–**K**) and littermate *Ext1f/f* mice (**H**, Ext1f/f) received tamoxifen injections at P5 and P7. (**A**–**G**) HE staining of coronal sections of *AcanCreER;Ext1f/f* mouse forelimbs. Palovarotene was administered every day at 3.5 weeks of age via gavage at doses of 0.26 mg/kg (**B**) or 1.76 mg/kg (**C**,**E**) for 2 (**B**,**C**) or 4 (**E**) weeks. One group received oral gavage of 1.76 mg/kg palovarotene for 2 weeks and then 4.0 mg/kg for an additional 2 weeks (**F**). Forelimbs were harvested at 5.5 weeks (**B**,**C**, 2 weeks of treatment) or 7.5 weeks (**E**,**F**, 4 weeks of treatment). Control mice were treated with corn oil after tamoxifen injections; their forelimbs were harvested at 5.5 (**A**) or 7.5 (**D**) weeks. Bars denote 200 μm. Measurements of volumes of osteochondromas (**G**, 3.5 weeks [starting of treatment, black; 5.5 weeks [2 weeks of treatment, blue; 7.5 weeks [4 weeks of treatment, red]). (**H**), The length of the ulna bones of control (blue)- and palovarotene (red)-treated mice was radiographically measured at 7.5 weeks of age. (**I**–**K**), NRX-204647-loaded (1 μg, 2 μL per limb, **I**) or control nanoparticles (**J**, 2 μL per limb) were injected in the distal epiphysis of the radius and ulna of *AcanCreER;Ext1f/f* mice twice a week starting at 3.5 weeks of age, 1 week after tamoxifen injections. The coronal view of the microCT images (**I**,**J**). The bar denotes 0.5 mm. The osteochondroma volume was measured (**K**, right, control, blue; left, NRX204647, red).

To determine whether palovarotene inhibited the growth of pre-existing osteochondromas, *AcanCreER;Ext1f/f* mice received tamoxifen injections at P5 and P7 and daily palovarotene gavage starting at 3.5 weeks of age when osteochondromas were visible (Figure 1A). A 2-week treatment with 1.76 mg/kg palovarotene strongly reduced tumor growth compared with the control group, whereas a 2-week treatment with 0.26 mg/kg palovarotene did not significantly reduce tumor growth (Figure 2A,B,G and Supplementary Table S2). Treatment with 1.76 mg/kg palovarotene inhibited tumor growth to a size similar to that at the beginning of treatment (Figure 2C,G and Supplementary Table S2; 3.5 weeks vs. 5.5 weeks, 1.76 mg/kg). When treatment with 1.76 mg/kg palovarotene was prolonged for an additional 2 weeks, osteochondromas were evident in both the ulna and radius of the forelimbs (Figure 2E), although the tumor volume was smaller than that in the control group (Figure 2D,E,G and Supplementary Table S2). To determine whether the weakening of antitumor action was rescued by increasing the dose, we changed the regimen to 1.76 mg/kg for 2 weeks, followed by 4 mg/kg for 2 weeks. This regimen strongly reduced the osteochondroma volume in both the ulna and radius of the forelimbs (Figure 2F,G and Supplementary Table S2). We did not observe a significant difference in tibia length between control and palovarotene-treated *AcanCreER;Ext1f/f* mice (Figure 2H and Supplementary Table S3, *Acan;Ext1f/f*). However, the palovarotene regimen resulted in a reduction in tibia length in *Ext1f/f* mice compared to that in the vehicle control (Figure 2H and Supplementary Table S3, *Ext1f/f*).

Given the in vivo data indicating that palovarotene injection reduced the tibia length in mice, we sought to determine whether the local injection of RARγ agonists can ameliorate skeletal toxicity. *AcanCreER;Ext1f/f* mice received tamoxifen injections at P5 and P7 and received an injection of nanoparticles containing 1 μg of the NRX204647 RARγ agonist (NRX-NP) using a micro-injector. The injections were delivered twice a week for 2 weeks in the right distal wrist. PTA-enhanced microCT analysis demonstrated that local injection of NRX-NPs inhibited osteochondroma growth (Figure 2I–K and Supplementary Table S4). However, extending the treatment for an additional two weeks with the same regimen failed to suppress tumor growth (Supplementary Figure S3A,C). NRX-NP treatment did not cause an imbalance in limb length in either *AcanCreER;Ext1f/f* or *Ext1f/f* mice, as determined by measurement of the ulna length (Supplementary Figure S3B,D).

### 2.3. Actions of Palovarotene on Osteochondromas

**Figure 3:**
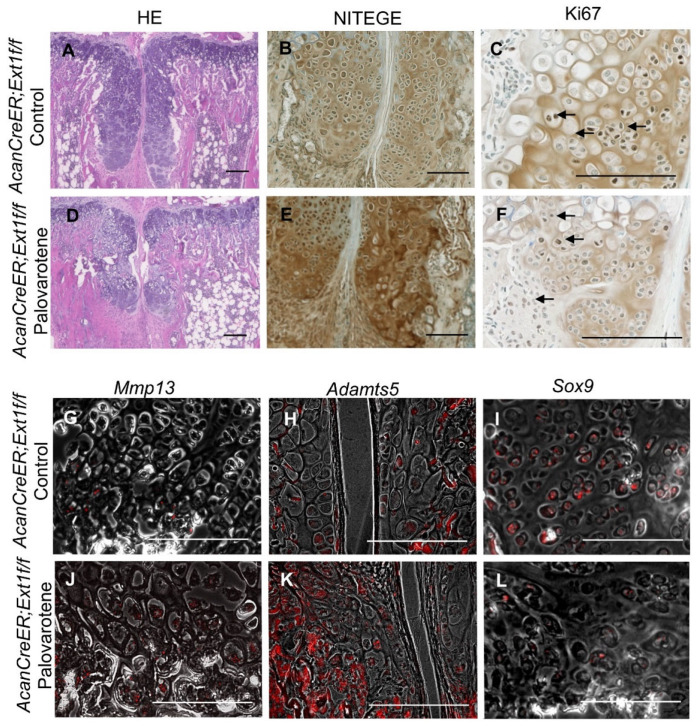
Histological analysis of palovarotene-treated osteochondromas. *AcanCreER;Ext1f/f* mice received palovarotene (1.76 mg/kg) (**D**–**F**,**J**,**K**) or corn oil (Control, **A**,**B**,**G**–**I**) gavage daily for 7 days, starting at 3.5 weeks of age after tamoxifen injections at P5 and P7. The forelimbs were then harvested. Coronal sections of the forelimbs were acquired and examined by staining with HE (**A**,**D**), immunostaining for NITEGE (**B**,**E**) or Ki67 (**C**,**F**, arrows indicate positively stained nuclei), or in situ hybridization for *Mmp13* (**G**,**J**), *Adamts5* (**H**,**K**), or *Sox9* (**I**,**L**) transcripts. Arrows indicate positively stained cells (**C,F**). The transcript (red) image is superimposed with the phase contrast image. (**H**,**J**–**L**). Bars denote 200 μm.

Palovarotene stimulates cartilage matrix degradation and inhibits cartilage matrix synthesis in human osteochondromas in explant cultures [14]. To determine whether the inhibition of osteochondroma growth by palovarotene in mice was associated with these inductive changes, osteochondroma sections were prepared from tamoxifen-injected *AcanCreER;Ext1f/f* mice one week after palovarotene or vehicle treatment. These two groups showed different histology. Vehicle-treated control osteochondroma showed a growth plate-like structure rotated at a 90° angle (Figure 3A). In contrast, palovarotene-treated osteochondroma tissues were divided into proximal and distal parts containing more hypertrophied cells than in the control group (Figure 3A vs. Figure 3D). The proximal portion of the palovarotene-treated osteochondroma was composed of smaller cells. Immunohistochemical staining with an antibody recognizing the C-terminal neoepitope of aggrecanase-cleaved aggrecan (NITEGE) revealed that the palovarotene-treated osteochondromas contained larger amounts of degraded aggrecan products than in the control group (Figure 3B vs. Figure 3E). The in situ hybridization demonstrated the upregulation of *matrix metalloproteinase 13* (*Mmp13*; Figure 3G vs. Figure 3J) and *a disintegrin and metalloproteinase with thrombospondin motifs 5* (*Adamts5*; Figure 3H vs. Figure 3K). The expression of *SRY-box transcription factor 9* (*Sox9*) was downregulated in the palovarotene-treated osteochondromas (Figure 3I vs. Figure 3L). Inhibition of cell proliferation activity by palovarotene was not evident, as determined using the immunohistochemistry of Ki67 (Figure 3C vs. Figure 3F, arrows). These results indicate that palovarotene stimulates matrix degradation in osteochondroma tissues, which may play an important role in inhibiting tumor growth.

### 2.4. Actions of Palovarotene on Cultured Chondrocytes

**Figure 4:**
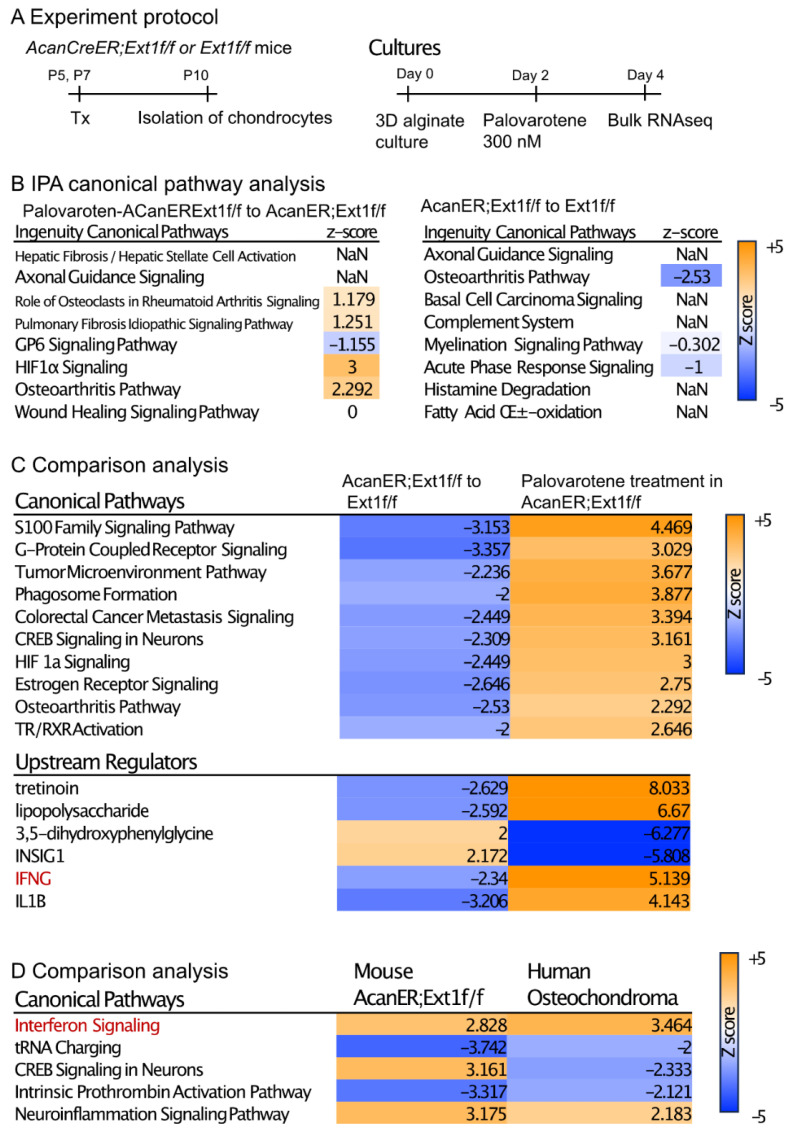
Transcriptome analysis of the effects of palovarotene on cultured chondrocytes. (**A**) Experiment protocol. Epiphyseal chondrocytes were isolated from *AcanCreER;Ext1f/f* and *Ext1f/f* mice at P10 after tamoxifen injections at P5 and P7. The isolated chondrocytes were seeded in alginate beads (40 μL) at a density of 0.5 × 10^6^ cells/mL. After 2 days, the cultures were treated with 300 nM palovarotene or the same volume of vehicle solution (ethanol) for 2 days. The cells were then dissociated and subjected to bulk RNA-seq analysis. (**B**) IPA of the DEGs was performed in control and palovarotene-treated chondrocyte cultures isolated from *AcanCreER;Ext1f/f* mice (**left**), and *AcanCreER;Ext1f/f* and *Ext1f/f* chondrocytes cultured without palovarotene treatment (**right**). The top eight pathways are shown. The osteoarthritis pathway was significantly activated in *AcanCreER;Ext1f/f* chondrocytes by palovarotene, while this pathway was significantly downregulated by *Ext1* deletion. (**C**) Heat maps of the comparison analysis of two conditions: *AcanCreER;Ext1f/f* vs. *Ext1f/f* chondrocytes (AcanER;Ext1f/f to Ext1f/f), and palovarotene-treated vs. untreated *AcanCreER;Ext1f/f* chondrocytes (Palovarotene treatment in AcanER;Ext1f/f). The top 10 and 5 ranked canonical pathways and upstream regulators, respectively, are shown. The opposite alterations caused by *Ext1* deficiency and palovarotene treatment were visualized. (**D**) Heat map of the comparative analysis of two conditions: palovarotene-treated vs. untreated *AcanCreER;Ext1f/f* mouse chondrocytes (Mouse AcanER;Ext1f/f), and palovarotene-treated vs. untreated human osteochondroma explants (Human Osteochondroma). Previously reported results of palovarotene treatment in human osteochondroma explants [14] were used. Similar differences, including activation of the interferon signaling pathway, were observed with these two conditions.

To understand the direct action of palovarotene on osteochondroma cells, we performed transcriptome analysis of cultured chondrocytes (Figure 4A). *AcanCreER;Ext1f/f* and *Ext1f/f* littermates received tamoxifen injections at P5 and P7, and epiphyseal chondrocytes were isolated from these mice at P10. The isolated cells were embedded in alginate beads, acclimated to culture for 2 days, and treated with 300 nM palovarotene or vehicle ethanol for 2 days. The cells were then released from the alginate gels and subjected to bulk RNA-seq. Differentially expressed genes (DEGs) were identified by both false discovery rate (FDR) <0.05 and log2 fold change with cutoff values of ±1 (Supplemental Figure S3A, GSE254042). A comparison between palovarotene-treated and control *AcanCreER;Ext1f/f* chondrocytes revealed that 2582 genes were significantly upregulated and 2222 genes were downregulated. A comparison between palovarotene-treated and control *Ext1f/f* chondrocytes demonstrated that 2629 and 2450 genes were significantly upregulated and downregulated, respectively (Supplementary Figure S4A). A comparison between *AcanCreER;Ext1f/f* and *Ext1f/f* chondrocytes and between palovarotene-treated *AcanCreER;Ext1f/f* and *Ext1f/f* chondrocytes revealed that a small number of genes were significantly altered, with 33 genes upregulated and 27 genes downregulated in *AcanCreER;Ext1f/f* chondrocytes compared to those in *Ext1f/f* chondrocytes, and 49 genes upregulated in palovarotene-treated *AcanCreER;Ext1f/f* chondrocytes compared to those in *Ext1f/f* chondrocytes. *Ext1* expression was significantly reduced in *AcanCreER;Ext1f/f* and palovarotene-treated *AcanCreER;Ext1f/f* (14.3% reduction, *p* = 0.00013, and 25.1% reduction, *p* = 7.59 × 10^−17^, respectively). Since full completion of Cre recombination in *Ext1^e2neofl^* mice is expected to reduce *Ext1* expression by 50% [15], the efficiency of recombination in *AcanCreER;Ext1f/f* chondrocytes by tamoxifen injections was considered to be 30–50%. Functional analysis of the DEGs was performed using Ingenuity Pathway Analysis (IPA) (Figure 4B, Supplementary Figure S4B, and Supplementary File). The DEGs in the palovarotene-treated *AcanCreER;Ext1f/f* chondrocytes compared to those in control *AcanCreER;Ext1f/f* chondrocytes were strongly linked to the upregulation of the hypoxia-inducible factor 1a (HIF1a) signaling and osteoarthritis pathways (Figure 4B). Canonical pathway analysis demonstrated that the DEGs in *AcanCreER;Ext1f/f* chondrocytes compared to *Ext1f/f* chondrocytes were highly linked to the downregulation of the osteoarthritis pathway.

Comparison analysis of two conditions (*AcanCreER;Ext1f/f* vs. *Ext1f/f* and palovarotene-treated *AcanCreER;Ext1f/f* vs. control *AcanCreER;Ext1f/f*) revealed that multiple pathways, including the osteoarthritis pathway, were altered in the opposite manner (Figure 4C, canonical pathways), suggesting that the genes linked to these biological pathways are important for both osteochondroma formation and palovarotene-induced inhibition of osteochondroma growth. The possible upstream regulators that drive the counteracting changes between *Ext1* deficiency and palovarotene treatment included retinoid drugs (tretinoin), inflammatory molecules (lipopolysaccharide, interferon-gamma [IFNG], and interleukin 1B [IL1B]), and metabolic regulators (3,5-dihydroxyphenylglycine and insulin-induced gene 1 [INSIG1]) (Figure 4C, upstream regulators). We performed a comparison analysis of the results obtained from palovarotene-treated *AcanCreER;Ext1f/f* cultured chondrocytes and palovarotene-treated human osteochondroma explants [14]. Activation of the interferon signaling pathway was observed in both palovarotene-treated cultures (Figure 4C,D, red).

### 2.5. Stimulation of the Stat3 Pathway by Palovarotene

**Figure 5:**
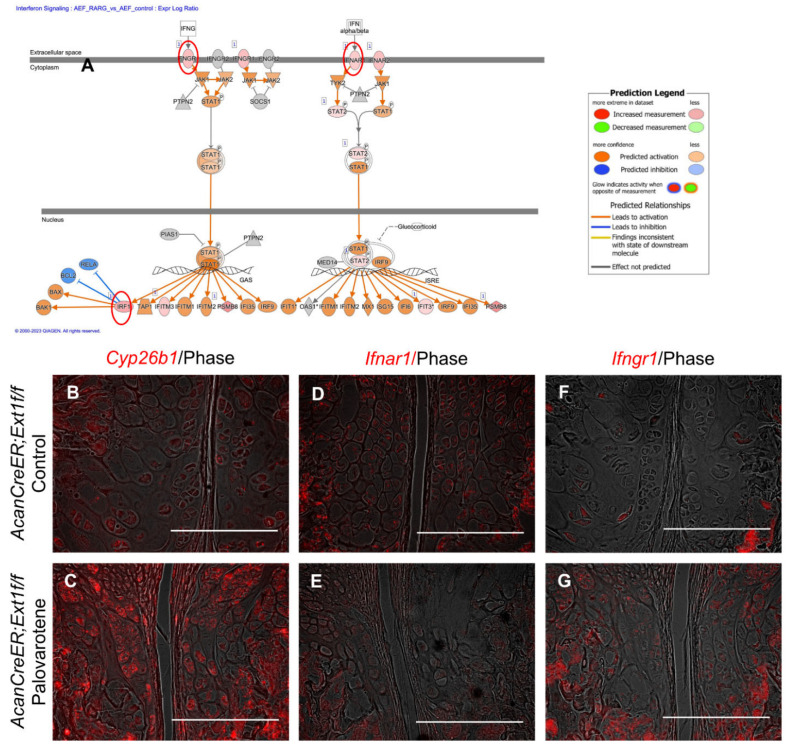
Palovarotene-induced changes in gene expression related to the interferon signaling pathway. (**A**) Interactive diagram for the altered interferon signaling pathway in palovarotene-treated *AcanCreER;Ext1f/f* chondrocytes. (**B**–**G**) *AcanCreER;Ext1f/f* mice received palovarotene (Palovarotene, 1.76 mg/kg) (**B**–**F**) or corn oil (Control) (**C**–**G**) gavage daily for 3 days starting at 3.5 weeks of age after tamoxifen injections at P5 and P7. The forelimbs were then harvested. Coronal sections of the forelimbs were acquired and used for in situ hybridization for *Cyp26b1* (**B**,**C**), *Ifnar1* (**D**,**E**), or *Ifngr1* (**F**,**G**). Bars denote 200 μm.

The link between palovarotene action and the interferon signaling pathway was further analyzed. Expressions of the upregulated genes listed in the interferon signaling pathway (*interferon alpha and beta receptor subunit 1* [*Ifnar1*], *Ifng receptor 1* [*Ifngr1*], and *interferon regulatory factor 1* [*Irf1*]; Figure 5A, red circles) were examined in palovarotene-treated osteochondromas. The expression of *cytochrome P450 family 26 subfamily member 1* (*Cyp26b1*), a genomic target molecule of the retinoid signaling pathway, was upregulated in the osteochondroma region in the periphery of the growth plate of palovarotene-treated *AcanCreER;Ext1f/f* mice (Figure 5B vs. Figure 5C), indicating that osteochondromas responds to palovarotene. Expression of *Ifnar1* was detected in osteochondromas (Figure 5D), and palovarotene treatment did not upregulate the expression level (Figure 5E). In contrast, the expression of *Ifngr1* was upregulated in palovarotene-treated osteochondromas (Figure 5F vs. Figure 5G). 

**Figure 6:**
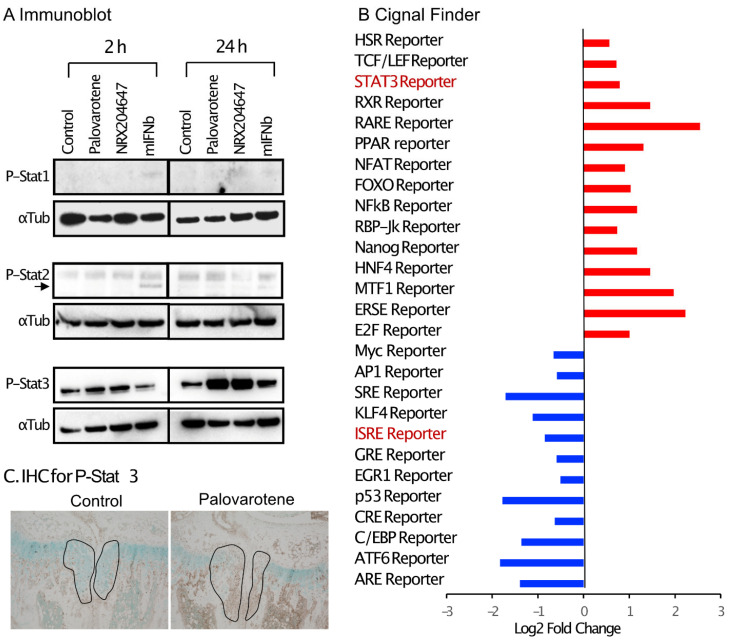
Palovarotene treatment stimulates the Stat3 pathway. (**A**) Immunoblot results of chondrocyte cultures. Primary epiphyseal chondrocytes were isolated from P3-P7 wild-type C57BL/6J mice and seeded at a density of 3.0 × 10^4^ cells/cm^2^ in high glucose Dulbecco’s modified Eagle’s medium containing 10% fetal bovine serum. On Day 2, the cultures were treated with 300 nM palovarotene, 100 nM NRX204647, or 150 ng/mL mouse interferon beta (mIFNb) for 2 or 24 h. The cultures were subjected to immunoblot analysis with antibodies to phospho-Stat1, phospho-Stat2, or phospho-Stat3, followed by reblotting with an anti-α- tubulin antibody. (**B**) Cignal Finder^®^ reporter assay data. Primary epiphyseal chondrocytes isolated from RARγ null mice were subjected to reverse transfection with the reporter constructs on the Cignal Finder 45-Pathway Reporter Array plate at a density of 3.0 × 10^4^ cells/cm^2^. The next day, the culture was treated with 100 nM NRX204647 for 24 h. (**C**) Immunohistochemical staining for phospho-Stat3. *AcanCreER;Ext1f/f* mice received palovarotene (Palovarotene, 1.76 mg/kg) or corn oil (Control) gavage daily for 3 days starting at 3.5 weeks of age after tamoxifen injections at P5 and P7. The forelimbs were then harvested. Coronal sections of the forelimbs were immuno-stained with the phospho-Stat3 antibody, followed by Fast Green counterstaining.

Interferon signaling is mediated by the phosphorylation of Stat1 (signal transducer and activator of transcription 1) and/or Stat2 [16]. Cultured chondrocytes were treated with palovarotene or mouse recombinant interferon beta protein (mIFNb) for 2 and 24 h, and cell lysates were subjected to immunoblotting. Slight stimulation of the phosphorylation of Stat1 and Stat2 was detected 2 h after the IFNb treatment, but not after 24 h. Palovarotene did not induce phosphorylation of Stat1 or Stat2 at either time point (Figure 6A). Phosphorylation of Stat3 is stimulated by the type I interferon signaling pathway [17]. Phosphorylation of Stat3 was strongly stimulated by palovarotene and IFNb 24 h after treatment (Figure 6A), suggesting that palovarotene stimulated the Stat3 pathway. Selective activation of the Stat3 pathway, but not Stat1 or Stat2, was confirmed by the results of a reporter assay using the Cignal Pathfinder. The Stat3 pathway was significantly stimulated (Figure 6B, STAT3 Reporter), while the Stat1 and Stat1/2 pathways were not (Figure 6B, GAS Reporter and ISRE Reporter, respectively). Palovarotene increased the STAT3 reporter activity in RARγ-hetero (RARγ+/−) chondrocytes, but not in RARγ-null chondrocytes (Supplementary Figure S5), indicating that Stat3 activation was specific to RARγ. Furthermore, palovarotene-treated osteochondromas contained more phospho-Stat3 (Figure 6C). These results indicate that palovarotene stimulates the Stat3 pathway.

## 3. Discussion

### 3.1. Clinically Relevant Regimen of Drug Treatment for Osteochondromas

Patients generally present to the clinic with pre-existing osteochondromas after they notice lumps made by growing cartilaginous tumors. In contrast, preclinical studies on osteochondroma treatment have used a regimen in which drug treatment begins at the onset or very early stage of tumor formation [7,8,12]. In this study, palovarotene treatment was initiated after osteochondromas developed to a certain level (Figure 1). This treatment stopped the increase in tumor volume (Figure 2), suggesting that palovarotene has an anti-tumor effect on pre-existing osteochondromas. The doses of palovarotene orally administrated in mice, 1.76 mg/kg and 4.0 mg/kg correspond to human equivalent doses of 0.14 mg/kg and 0.32 mg/kg, respectively, according to the practice guide on dose conversion between mice and humans. [18] These converted doses are comparable to the flare-up doses of palovarotene (SOHONOS) for patients with FOP (0.125 to 0.5 mg/kg, https://www.ipsen.com/websites/Ipsen_Online/wp-content/uploads/sites/61/2024/01/05103949/PM-Sohonos-EN-17Nov2023.pdf, accessed on 27 June 2024).

Palovarotene treatment did not result in complete tumor regression, and the dose of palovarotene had to be increased to achieve complete tumor suppression over time (Figure 2). Histological analyses indicated that the mechanism of tumor inhibition involved the destruction of cartilaginous tissue by degradation of the cartilage matrix and stimulation of chondrocyte hypertrophy rather than by the suppression of the proliferation of osteochondroma cells (Figure 3). The findings indicate that combined treatment with anti-proliferative drugs may have better therapeutic effects. To date, no serious adverse events, including early growth plate closure, have been reported in a clinical study of multiple osteochondromas (MO) (NCT03442985). However, this clinical trial was placed on hold because of the toxicities observed with chronic dosing in the FOP trial [10]. Because the aforementioned MO study was primarily a pediatric study with an upper age of enrollment of 14 years, a period of life when skeletal growth is active, this discontinuation was unavoidable. We observed that increasing the dose of palovarotene required to maintain the long-term suppression of tumor growth negatively affected skeletal growth in mice (Figure 2), suggesting that high doses and long-term administration of the RARγ agonist in this mouse model have adverse effects on skeletal cells such as chondrocytes and osteoblasts. Site-specific delivery in combination with controlled drug release could be beneficial by enhancing therapeutic effects and reducing the toxicity associated with systemic drug administration. Local drug delivery using nanocarriers has been shown to both improve efficacy and minimize side effects on non-target tissues [19]. Nanocarrier-based formulations can not only deliver drugs to their local sites of action but also protect labile compounds from premature degradation, increasing and sustaining drug bioavailability. As an experimental approach for suppressing the localized disease while minimizing systemic skeletal toxicity, we tested the nanoparticle-based local delivery of a RARγ agonist. NRX-NP significantly inhibited tumor growth (Figure 2), with our results demonstrating the feasibility of nanoparticle-based drug delivery, potentially paving the way to the development of a safer and more effective, site-specific drug therapy for osteochondromas. However, the inhibition efficacy was less (59% inhibition) than that of the systemic administration (88% inhibition) in the 2-week treatment and faded in the 4-week treatment (Figure 2 and Supplementary Figure S3). Previously, we succeeded in inhibiting the growth of xenografts of well-differentiated human chondrosarcoma cells over 7 weeks using the same NRX-NP [20]. The difference in the effectiveness of the NRX-NP in these two systems can be attributed to several factors. The tested human chondrosarcoma cell xenograft was more sensitive to RARγ agonists than that were mouse osteochondromas. The anatomical location of osteochondromas surrounding the bones makes it difficult to target tumor cells and retain nanoparticles for a long period. Finally, the location of the xenograft in the middle of the back made it easy to apply a large amount of NRX-NP. Improvements in drug delivery systems and application techniques will be the future direction of this project.

### 3.2. Actions of Palovarotene on Chondrocytes

RARγ plays an important role in the regulation of cartilage development [14]. RARγ is dominantly expressed in cartilage compared to other RAR family members, RARα and RARβ. RARγ expression starts at the time of the appearance of cartilaginous skeletons in embryos [21]. RARγ mostly exists as a ligand-free form and supports the expression and accumulation of cartilage matrix, including sulfated proteoglycans and type II collagens [21,22]. Retinoic acid treatment of limb buds and chondrocytes strongly inhibits the synthesis of proteoglycans and type II and IX collagens [23,24,25]. These inhibitory effects of retinoid signaling on chondrogenesis and cartilage matrix formation have been considered to be the mechanism of the antitumor action of palovarotene on osteochondromas; the inhibition of aberrant bone morphogenetic protein signaling by retinoid signaling has been addressed [9,26]. The stimulation of retinoid signaling by deletion of Cyp26b1, a responsible enzyme for retinoic acid degradation, in the growth plate inhibits chondrocyte proliferation and skeletal growth [27], indicating that silencing of retinoid signaling, presumably via RARγ, is required for proliferation of the growth plate chondrocytes. Hence, the stimulation of retinoid signaling by palovarotene was expected to inhibit chondrocyte proliferation in osteochondromas. However, this effect was not observed in our system.

Histological examination demonstrated that palovarotene stimulated the degradation of the cartilage matrix and inhibited the expression of *Sox9*, a master gene for cartilage matrix synthesis, leading to the destruction of the developing cartilaginous mass. RNA-seq analysis of cultured chondrocytes revealed the biological pathways altered by palovarotene treatment (Figure 4). Among them, the upregulation of the osteoarthritis pathway is the most relevant explanation for our observations. The genes enriched in the osteoarthritis pathway include the upregulation of various matrix degradation enzymes, such as *Adamts4*, *Adamts5*, *Mmp9*, and *Mmp13*, and the downregulation of cartilage-related genes, such as *collagen type II alpha 1 chain Col2a1*, *frizzled-related protein* (*Frzb*), *Indian hedgehog* (*Ihh*), and *Sox9* (Supplemental File, osteoarthritis pathway sheet). Changes in the expression of these molecules are believed to mediate disruption of the osteochondroma matrix. We observed that the osteoarthritis pathway was downregulated in chondrocytes isolated from tamoxifen-injected *AcanCreER;Ext1f/f* mice compared to those isolated from tamoxifen-injected *Ext1f/f* mice (Figure 4). Although the extent of deletion of the *Ext1* gene was mild, downregulation of the osteoarthritis pathway may be responsible for the mechanism of osteochondroma formation in *AcanCreER;Ext1f/f* mice. The HIF1α signaling pathway plays an important role in cartilage development [28] and is up-regulated in embryonic developing cartilage [29]. Hypoxia stimulates HIFα signaling and promotes the expression of the chondrogenic phenotype in culture [30]. The finding that palovarotene upregulated the HIF1α signaling pathway in chondrocytes while decreased expression of *Ext1* downregulated the HIF1α signaling pathway (Figure 4) seems contradictory to the current understanding of this pathway in the regulation of cartilage development. However, the genes enriched in this pathway identified by IPA were related to matrix degradation (upregulation of *Mmps*) and angiogenesis (upregulation of *Vegfs*). These events have been caused by molecular changes during hypoxia-induced metastasis of tumor cells [31]. In addition, chondrogenic genes were hardly upregulated in this pathway (Supplemental File, HIF1a pathway sheet).

RNA-seq data analysis using IPA revealed the upregulation of the interferon signaling pathway in both palovarotene-treated mouse chondrocytes and palovarotene-treated human osteochondroma explants (Figure 4). The relationship between interferons and retinoids has been previously studied in cancer [32]. The combination of interferons and retinoids potentiates antitumor action in certain types of cancer cells, such as leukemia, mammary carcinoma, and squamous carcinoma cells. Although the molecular basis for the synergistic antitumor action of interferons and retinoids is not completely understood, studies with anti-sense-based knockout genetic screens have revealed the importance of family genes associated with retinoic acid and interferon-induced cell death, which are termed genes associated with retinoid-interferon-induced mortality (GRIM) [32]. However, in palovarotene-treated chondrocytes, the *GRIM-1* (*Shq1*), *GRIM-12* (*Txnrd1*), and *GRIM-19* (*Ndufa13*) genes, which have been studied among 12 genes [33], were not significantly upregulated. The results of in situ hybridization were not always consistent with the RNA-seq results; upregulation of *ifngr1* was observed in the palovarotene-treated osteochondroma in vivo, whereas upregulation of *ifnar1* was not (Figure 5). Palovarotene stimulated Stat3 phosphorylation at Y405 in chondrocytes but not Stat1 or Stat2 phosphorylation (Figure 6). The reporter assay demonstrated that the RARγ agonist stimulated the Stat3 pathway but not the Stat1 and Stat1/2 pathways (Figure 6). Stat1 and Stat 2 are considered dominant mediators of the type I interferon signaling pathway, but Stat 3 is not an indispensable regulator and is involved in the fine balance of interferon signaling as a negative or positive regulator [17]. The association between retinoid signaling and interferon signaling observed by transcriptome analysis may be specific to the Stat 3 pathway but not via crosstalk with a typical interferon pathway.

Stat3 is recognized as an acute downstream intracellular effector of inflammatory cytokines and growth factors, including IL-6 and EGFs, and is involved in the regulation of DNA transcription [34]. Activation of Stat3 is controlled by the phosphorylation of Tyr705 and Ser727 of its Src homology domain. The involvement of the Stat3 pathway in the regulation of skeletal tissue homeostasis has recently received attention [35]. Liang et al. [36] reported that upregulation of Stat3 phosphorylation (Tyr 705) was observed in osteoarthritic articular cartilage in human samples and a surgery-induced osteoarthritis mouse model. In this study, the antagonist of the retinoic acid receptor-related orphan receptor-alpha (RORα) inhibits the IL6-Stat3 pathway in cultured human chondrocytes, but it remains unclear if RORα can activate the Stat3 pathway. Many independent studies have demonstrated the involvement of Stat3 signaling activation in degenerative changes in articular cartilage in osteoarthritis [37]. Actions of the Stat3 pathway in the context of osteoarthritis pathology include the stimulation of matrix degradation and cell death [38,39]. The genes enriched in the osteoarthritis pathway revealed by IPA of the DEGs in the palovarotene-treated chondrocytes included various matrix catabolic genes (*Adamts4*, *Adamts5*, *Mmp9*, *Mmp10*, *Mmp12*, and *Mmp13*) and cell death-related genes (*Casp1*, *Casp3*, and *Casp6*). *Adamts4*, *Mmp9*, *Mmp13*, *Casp3*, and *Casp6* are target genes of Stat 3 (Harmonizezome 3.0, https://maayanlab.cloud/Harmonizome/, accessed on 16 February 2024, [40]). Taken together, our findings suggest that palovarotene stimulates the Stat3 signaling pathway and that the Stat3 pathway may underlie the antitumor action of palovarotene on osteochondromas, as this pathway is involved in cartilage degeneration in osteoarthritis.

## 4. Materials and Methods

### 4.1. Animals

All procedures were conducted following the National Institutes of Health guidelines of the Institutional Animal Care and USE Committee of the University of Maryland, Baltimore (protocols 0317003, 0120002, 0920002, and/or 00000311). *AggrecanCre^ERT2^* (JAX019148), *Ext1^e2neofl^* (*Ext1fl*, JAX009326), and *Rosa26-CAG-loxP-stop-loxP-ZsGreen* (*Ai6:R26R-ZsG*, JAX007906) mice were obtained from Jackson Laboratory (Bar Harbor, MN). We crossed *AggrecanCre^ERT2^* mice with *R26R-ZsG* and *Ext1^e2neofl^* to create *AggrecanCre^ERT2^;R26R^ZsGreen^* (*AcanCre^ERT2^;R26R^ZsGreen^*) and *AggrecanCre^ERT2^; Ext1^e2neofl^* (*AcanCreER;Ext1f/f*) mice. To achieve Cre recombination, 40 mg/kg body weight of tamoxifen (T5648; Sigma-Aldrich, St. Louis, MO, USA) dissolved in corn oil (C8267; Sigma-Aldrich) was intraperitoneally injected twice at P5 and P7. RARγ-deficient mice [41] were kindly provided by Dr. Pierre Chambon (INSERM, Paris, France). Animals were housed in a University Laboratory Animal Resources supervised animal facility with a 12-h light/dark cycle in a temperature (22 ± 1 °C) and humidity (55 ± 5%) controlled room. The animals were provided with hygienic animal bedding, and all cages contained wood shavings, bedding, and cotton pads. The health status of each animal was monitored throughout the experiments by investigators, animal veterinary technicians, and veterinarians, according to institutional guidelines. The mice were free from viral, bacterial, and parasitic pathogens during the experimental period.

### 4.2. Drug Treatment

Atomax Chemicals Co., Ltd. (Shenzhou, China) prepared two selective RARγ agonists (palovarotene and NRX204647). The company synthesizes chemicals as described in their respective patent applications (WO 2001009076 for palovarotene) with over 98% purity. Once we received the products, their quality and biological activity were verified using nuclear magnetic resonance and mass spectrometry, and the reporter assay using the HEK293 cells that are stably integrated contained a firefly luciferase gene under the control of retinoic acid response elements along with the full-length human *RARG* gene (BPS Bioscience, San Diego, CA, USA). The drug powder was stored at −30 °C under desiccated conditions until use. Stock solutions were kept in an amber tube with argon gas and stored at −30 °C. The prepared working solution was sufficient for a set of experiments, dispensed in tubes for one-time use, and kept at −30 °C.

*AcanCreER;Ext1f/f*, *Ext1f/f*, or *AcanCre^ERT2^;R26R^ZsGreen^* mice received intraperitoneal injections of tamoxifen in corn oil/10% ethanol (40 mg/kg) at ages P5 and P7. At 3.5 weeks post-injection, *AcanCreER;Ext1f/f,* and *Ext1f/f* mice were sedated and received daily oral gavage of palovarotene or vehicle (corn oil). The treatment periods were 3 days, 1 week, 2 weeks, and 4 weeks (n = 3–6). Doses were 0.26 or 1.76 mg/kg of palovarotene or vehicle for 2 or 4 weeks, and 1.76 of mg/kg palovarotene or vehicle for 2 weeks, followed by 4.0 mg/kg of palovarotene or vehicle for an additional 2 weeks. Because the manifestation of tumors was clearer in male mice than in female mice (Supplemental Figure S2A), male mice were used for the drug treatment experiments.

For local treatment, based on the chemical structure and receptor binding selectivity, NRX204647 was chosen as the RARγ agonist to be administered in PLA-based NPs (NRX-NP). NP was formulated by modifying the emulsification-solvent evaporation method using serum albumin as a colloidal stabilizer [42]. Briefly, NRX204647 (15 mg) was co-dissolved in 200 mg PLA (Mn = 19 kDa, polydispersity index = 1.8; Birmingham Labs, Birmingham, AL, USA) in chloroform (5 mL). The organic phase was emulsified by sonication in an aqueous solution containing 150 mg of human serum albumin (Octapharma AB, Stockholm, Sweden) dissolved in 10 mL of deionized water, and the organic solvent was removed using rotary evaporation under reduced pressure. NP was passed through a sterile 5.0-µm membrane, lyophilized with glucose (5% *w*/*v*) as a cryoprotectant, stored at −80 °C, and reconstituted in deionized water before use. The drug loading of NP was determined spectrophotometrically following extraction in *sec*-butanol against a suitable calibration curve. Measurements of NP sizes were performed using dynamic light scattering. NRX-NP (0.5 μg/1 μL at each site) was subcutaneously injected at the medial and lateral sites of the vicinity of the proximal growth plate of the radius and ulna of the mice injected with tamoxifen (40 mg/kg) at P5 and P7 twice a week for 2 weeks and 4 weeks, beginning at 3.5 weeks of age. At each endpoint, the forelimbs were harvested and processed for X-ray, histological, microCT, or in situ hybridization analysis.

### 4.3. Histology and Immunohistochemical Staining

Tissues for histological examination were fixed with 4% paraformaldehyde (PFA) for 24 h at 4 °C. Samples were decalcified with 10% ethylenediaminetetraacetic acid at pH 7.2 after capturing radiographic images. The samples were processed for paraffin embedding. Sagittal sections of 5-μm in thickness were created throughout the project. Sections were stained with HE and safranin O. For immunohistochemical staining, the sections were baked for 1 h at 60 °C and then deparaffinized. Sections (n = 2–4) were incubated in 3% hydrogen peroxide in methanol for 10 min. Sections were then incubated with 400 IU/mL hyaluronidase for 30 min at 37 °C (NITEGE) or 10 mM sodium citrate buffer solution (pH 6.0) for 8 min at 80 °C (Ki67 and phosphor-Stat3), followed by blocking buffer, 5% bovine serum albumin, and 1% goat serum for 60 min at room temperature. The sections were then incubated with the primary antibody against aggrecan degradation products (NITEGE, 1:3000; provided by J. Mort, Shriners Hospital for Children, Montreal, Quebec, Canada), Ki67 (1:300, ab16667; Abcam, Boston, MA, USA), or phospho-Stat3 (1:1000, 9145; Cell Signaling Technology, Beverly, MA, USA) overnight at 4 °C. Sections were then incubated with a 1:200 dilution of biotinylated anti-rabbit IgG (Vector Laboratories, #BA-1000-1.5, Burlingame, CA, USA), followed by color detection using Vectastain Elite ABC HRP reagent RTU (Vector Laboratories, #PK-7100) and counterstaining with methyl green (Alfa Aeser, Haverhill, MA, USA). The results were observed and captured using a BZX700 microscope (Keyence, Itasca, IL, USA).

### 4.4. Tumor Volumetric Analysis by PTA-Enhanced microCT

To establish specific protocols for PTA-enhanced microCT that allow microlevel visualization of cartilage tissues, including the growth plate and osteochondroma, we adapted and expanded a previously described protocol [43]. Samples were immersed in 2% KOH for 3–7 days after fixation with 4% PFA overnight. Under a dissection microscope, the distal forelimb was exposed by removing tendons and soft tissues. Forelimbs were then incubated in 0.7% PTA (CAT: 12501-23-4; Sigma-Aldrich) in 70% ethanol at room temperature for one week. Samples were placed in 70% ethanol without PTA and scanned using a Skyscan 1172 microCT scanner (Bruker, Billerica, MA, USA). Image acquisition at a resolution of 6.5 µm/pixel required 45 min (parameters: 0.2 mm Al filter using a 55 kV X-ray source, resolution at 2 K, step rotation of 0.51, and two average frames using 360° rotation). Images were reconstructed using NRecon software (version 1.7.4.6, Bruker). The cartilage tumor area was manually selected, and the tumor volume was analyzed.

### 4.5. In Situ Hybridization by RNAscope

The forelimbs were fixed with 2% formaldehyde, dehydrated by increasing the sucrose percentage to 30%, and then embedded in the optimal cutting temperature (O.C.T.) compound (Thermo Fisher Scientific, Waltham, MA, USA). Undecalcified frozen sections were mounted on Kawamoto’s film and subjected to in situ hybridization using the RNAscope^®^ Fluorescent Multiplex Kit (Advance Cell Diagnostic Inc., Newark, CA, USA). Predesigned single- or multiplex probes (*Cyp26b1*, *Mmp13*, *Adamts5*, *Sox9*, *Infar1*, *Infgr1*, and *Itf1*) were obtained from the same company.

### 4.6. Chondrocyte Culture

Primary mouse chondrocytes were isolated from *AggCreER;Ext1f/f and Ext1f/f* mice at P8 after injections of tamoxifen (40 mg/kg) at P5 and P7, using a previously reported protocol [44]. The isolated cells were embedded in 1.2% alginate (Cosmo Bio USA, Carlsbad, CA, USA) at a density of 0.5 × 10^6^/mL following the manufacturer’s protocol and cultured in high glucose DMEM containing 10% fetal bovine serum under a 3% O_2_ atmosphere. On day 2, the cultures were treated with 300 nM palovarotene or an equal volume of vehicle ethanol for 2 days. The cells were then released from alginate by incubation with 0.055 M sodium citrate, 0.03 M EDTA, and 0.15 M NaCl pH 6.8 for 5 min at 4 °C and lysed in RLT buffer (Qiagen, Redwood City, CA, USA).

### 4.7. Transcriptome Analysis

Total RNA preparation, library preparation, and RNA-seq analysis were performed at the Maryland Genomics Institute for Genome Sciences, University of Maryland School of Medicine. Total RNA was extracted from the cultured chondrocytes using the RNeasy Mini Kit (Qiagen, Hilden, Germany). Strand-specific, dual-unique indexed libraries for sequencing on all Illumina platforms were made using the NEBNext^®^ Ultra™ II Directional RNA Library Prep Kit for Illumina^®^ (New England Biolabs, Ipswich, MA, USA). The manufacturer protocol was modified by diluting adapter 1:30 and using 3 μL of this dilution. Library size selection was performed using AMPure SPRI-select beads (Beckman Coulter Genomics, Danvers, MA, USA). Glycosylase digestion of the adapter and second strand was performed in the same reaction as the final amplification to avoid further cleanup. Sample input for this method was polyA-enriched mRNA using the NEBNext^®^ Poly(A) mRNA Magnetic Isolation Module, a bead-based enrichment kit. Libraries were assessed for concentration and fragment size using a DNA High Sensitivity Assay on a LabChip GX Touch (Perkin Elmer, Waltham, MA, USA). Library concentrations were assessed using qPCR by the KAPA Library Quantification Kit (Complete, Universal) (Kapa Biosystems, Woburn, MA, USA).

RNAseq analysis was carried out by Maryland Genomics, Institute for Genome Sciences, UMSOM. Paired-end Illumina libraries were mapped to the mouse reference, Ensembl release GRCm39.109, using HiSat2 v2.1.0, using default mismatch parameters. The DESeq2 Bioconductor package (v1.5.24) was used to estimate dispersion, normalize read counts by library size to generate the counts per million for each gene, and determine differentially expressed genes between disease and control samples. Differentially expressed transcripts with a *p*-value ≤ 0.05 and log_2_ fold change ≥ 1 were used for downstream analyses. Normalized read counts were used to compute the correlation between replicates for the same condition and compute the principal component analysis for all samples. Pathway analysis was performed using IPA software (version 111725566, Qiagen). The data were deposited in the Gene Expression Omnibus database (GSE254042).

### 4.8. Immunoblot Protocol

Epiphyseal chondrocytes were isolated from P3-P7 C57BL/6J (JAX, 000664), plated on type I collagen-coated 60 mm dishes at a density of 2.0 × 10^6^ cells/cm^2,^ and cultured in high glucose DMEM containing 10% fetal bovine serum. After the cells grew to confluency, the medium was removed and replaced by high glucose DMEM containing 2% charcoal-treated serum with 300 nM palovarotene, 100 nM NRX204647, and 150 ng/mL mouse recombinant interferon beta (PBL Assay Science, Piscataway, NJ, USA) or 0.1% ethanol. Twenty-four hours after treatment, the cultures were lysed in RIPA buffer containing protease inhibitors, and the supernatants were stored at −80 °C until use. Cell lysates were subjected to SDS-PAGE (4–12%, NuPage Bis-Tris, Thermo Fisher Scientific) followed by western blotting with antibodies against phospho-Stat1 (7649), phospho-Stat2 (4441), and phosphor-Stat3 (9145) (all from Cell Signaling Technology). The antibodies bound to the membrane were visualized using the VECTASTATIN Elite Universal PLUS kit, peroxidase (Vector Laboratories), and SuperSignal West Femto (Thermo Fisher Scientific). The membranes were reblotted with an α-tubulin antibody (Thermo Fisher Scientific).

### 4.9. Reporter Assay

Primary epiphyseal chondrocytes isolated from RARγ null or hetero mice were subjected to reverse transfection with the reporter constructs on the Cignal Finder 45-Pathway Reporter Array plate (Qiagen) at a density of 3.0 × 10^4^ cells/cm^2^ following the manufacturer’s protocol, except for the use of Lipofectamine 3000 (Thermo Fisher Scientific) instead of Attractene. The next day, the cells were treated with 100 nM NRX204647 for 24 h. Luciferase assay was performed using the Dual-Glo Luciferase Assay System (Promega, Madison, WI, USA).

### 4.10. Statistics

The results were analyzed using Prism 10 version 10.0.1 (GraphPad Software, Boston, MA, USA). To compare two groups, Mann–Whitney U test was used. To compare multiple groups, Kruskal–Wallis test was used. The threshold for significance for all tests was set at *p* < 0.05. The relevant descriptive statistics were shown in Supplementary Tables S1–S4.

## Data Availability

All data supporting the findings of this study are available within the paper and its Supplementary Information. The RNA-seq data were deposited in the GEO database (GSE254042).

## Supplementary Materials

The following supporting information can be downloaded at https://www.mdpi.com/article/10.3390/ijms25147610/s1.
