# Supplementary material for: Analysis of the Actions of RARγ Agonists on Growing Osteochondromas in a Mouse Model
Source: Int J Mol Sci. 2024 Jul 11;25(14):7610. doi: 10.3390/ijms25147610 (PMC11277217; doi:10.3390/ijms25147610)
Supplement: Supplementary file 1 [file ijms-25-07610-s001.zip › ijms-3081768-supplementary-Supplementary figures and tables_Garcia.pdf]

Supplementary table S1. Descriptive statistics of comparison of the tumor volume at different ages shown in Figure 1J.

	3.5 weeks	5.5 weeks	7.5 weeks
Number of values	6	6	6
25% Percentile	0.240	0.645	0.890
Median	0.260	0.671	1.04
75% Percentile	0.295	0.751	1.24
IQR	0.0549	0.106	0.347
Mean	0.267	0.690	1.03
Std. Deviation	0.031	0.050	0.230
P value	3.5 weeks vs. 5.5 weeks		0.02
	3.5 weeks vs. 7.5 weeks		<0.001
	5.5 weeks vs. 7.5 weeks		0.19

Mean and standard deviation, quartiles, and P-value examined by Kruskal-Wallis test between groups are shown.

Supplementary table S2. Descriptive statistics of comparison of tumor volumes with or without palovarotene treatment for 2 (5.5 weeks) and 4 weeks (7.5 weeks) shown in Figure 2G.

	3.5 weeks	5.5 weeks			7.5 weeks		
	Control	Control	0.26 mg/Kg	1.76 mg/Kg	Control	1.76 mg/Kg	1.76-4 mg/Kg
Number of values	6	6	3	6	6	3	3
25% Percentile	0.241	0.645	0.637	0.161	0.89	0.661	0.276
Median	0.26	0.671	0.637	0.166	1.04	0.677	0.298
75% Percentile	0.295	0.751	0.637	0.202	1.24	0.712	0.369
IQR	0.054	0.106	0	0.041	0.35	0.051	0.093
Mean	0.267	0.689	0.637	0.178	1.03	0.684	0.314
Std. Deviation	0.031	0.050	0.0005	0.024	0.23	0.026	0.049

P value	Control 3.5 weeks vs. Control 5.5 weeks	0.013
	Control 3.5 weeks vs. 0.26 mg/Kg 5.5 weeks	0.24
	Control 3.5 weeks vs. 1.76 mg/Kg 5.5 weeks	0.24
	Control 3.5 weeks vs. Control 7.5 weeks	<0.001
	Control 3.5 weeks vs. 1.76 mg/Kg 7.5 weeks	0.036
	Control 3.5 weeks vs. 1.76 and 4 mg/Kg 7.5 weeks	0.66
	Control 5.5 weeks vs. 0.26 mg/Kg 5.5 weeks	0.39
	Control 5.5 weeks vs. 1.76 mg/Kg 5.5 weeks	<0.001
	Control 7.5 weeks vs. 1.76 mg/Kg 7.5 weeks	0.54
	Control 7.5 weeks vs. 1.76 and 4 mg/Kg 7.5 weeks	0.023
	1.76 mg/Kg 7.5 weeks vs. 1.76 and 4 mg/Kg 7.5 weeks	0.15

Mean and standard deviation, quartiles and P-value examined by Kruskal-Wallis test between groups are shown.

Supplementary Table S3. Descriptive statistics of comparison of the length of tibia bones between control and palovarotene (1.76 mg/Kg for 2 weeks and 4.0 mg/Kg for additional 2 weeks) in Ext1f/f and AcanCreER;Ext1f/f mice, shown in Figure 2H.

	Ext1f/f		*AcanCreER;Ext1f/f*	
	Control	Palovarotene	Control	Palovarotene
Number of values	8	4	4	4
25% Percentile	23.93	20.54	21.13	20.83
Median	24.66	21.32	21.3	21.25
75% Percentile	25.66	22.4	21.82	21.5
IQR	1.73	1.86	0.69	0.67
Mean	24.8	21.42	21.42	21.19
Std. Deviation	0.88	1.01	0.39	0.36
P value	*Ext1f/f* vs *AcanCreER;Ext1f/f*			
	Control		0.0427	
	Palovarotene		>0.999	
	Control vs Palovarotene			
	Ext1f/f		0.035	
	AcanCrer:Ext1f/f		>0.999	

Mean and standard deviation, quartiles, and P-value examined by Kruskal-Wallis test between groups are shown.

Supplement Table S4. Descriptive statistics of comparison of tumor volumes with or without NRX-NP injections for 2 shown in Figure 2K.

	Right	Left_drug
Number of values	5	5
25% Percentile	0.405	0.154
Median	0.53	0.294
75% Percentile	0.619	0.342
IQR	0.214	0.188
Mean	0.516	0.257
Std. Deviation	0.112	0.099
P value	0.008	

Mean and standard deviation, quartiles, and P-value examined by Mann-Whitney U test between groups are shown.

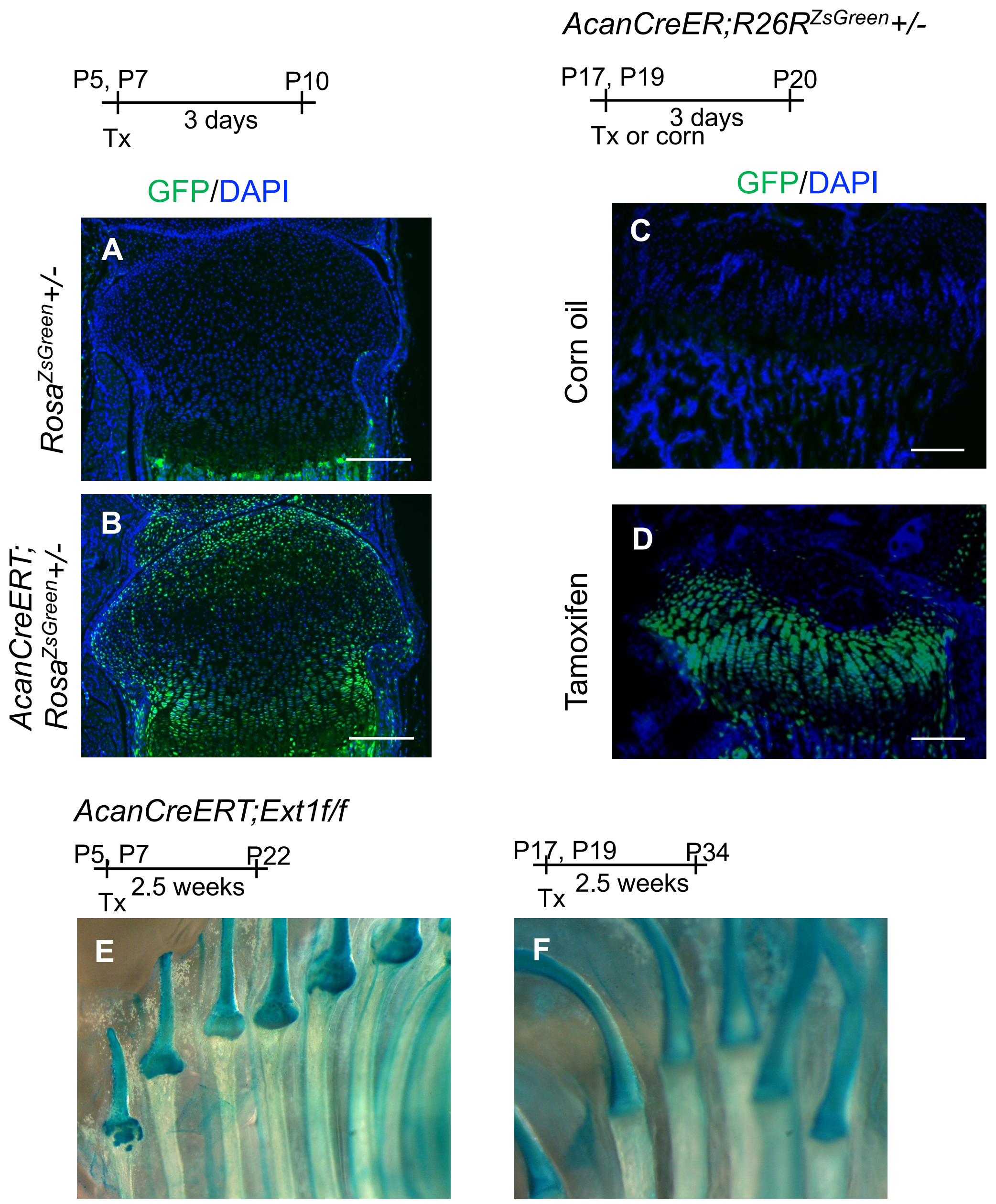

Supplementary Figure S1. Differences in the degree of tumor induction depend on the timing of tamoxifen administration. A, *AcanCreER;R26R*^{ZsGreen}+/- and *R26R*^{ZsGreen}+/- mice received tamoxifen injections at P5 and P7 (A and B, respectively), or P17 and P19 (D). *AcanCreER;R26R*^{ZsGreen}+/- mice received corn oil injections at P17 and P19 as control (C). The tibia was harvested at P10 or P20, 3 days after the initial tamoxifen or corn oil injection. The distribution of reporter proteins was visualized by detecting ZsGreen protein fluorescence and 4′,6-diamidino-2-phenylindole staining the nuclei of cells. Bars denote 200 µm. E and F, *AcanCreER;Ext1f/f* mice received tamoxifen injections at P5 and P7 (E), or P17 and P19 (F). The ribs were harvested at P22 or P34, 2.5 weeks after the initial tamoxifen injection, and stained with Alcian blue to visualize the cartilage.

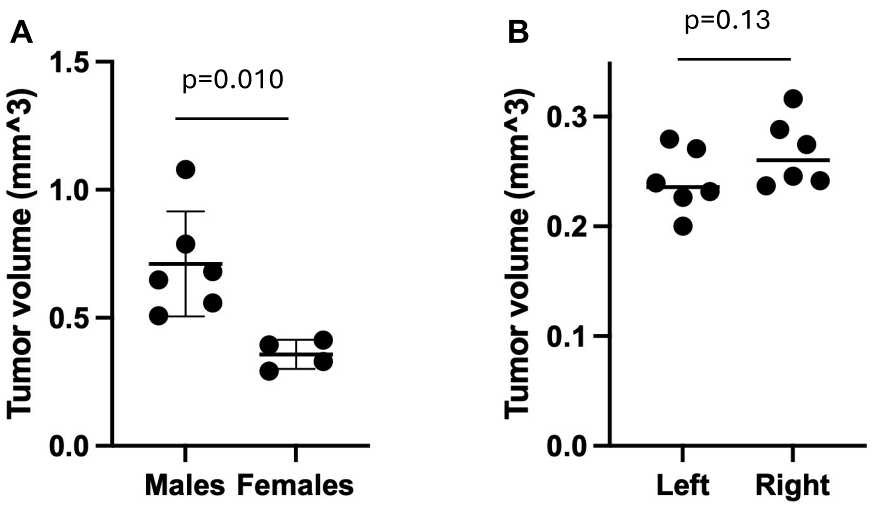

C

	Males	Females
Number of values	6	4
25% Percentile	0.545	0.301
Median	0.665	0.362
75% Percentile	0.861	0.409
IQR	0.316	0.108
Mean	0.711	0.357
Std. Deviation	0.206	0.057
P value	0.010	

D

	Left	Right
Number of values	6	6
25% Percentile	0.22	0.241
Median	0.236	0.26
75% Percentile	0.273	0.295
IQR	0.053	0.054
Mean	0.241	0.267
Std. Deviation	0.030	0.031
P value	0.13	

Supplementary Figure S2. Comparison of the degree of tumor formation between males and females and between left and right forelimbs. A, *AcanCreER;Ext1f/f* male (n = 5) and female (n = 4) mice received tamoxifen injections at P5 and P7. Forelimbs were harvested at 5.5 weeks of age. Serial coronal sections were prepared. The tumor area of each section was manually measured, and the total tumor volume was calculated by multiplying the sum of the tumor area by the section thickness (5 μm). B, *AcanCreER;Ext1f/f* male (n = 6) mice received tamoxifen injections at P5 and P7. The forelimbs were harvested at 3.5 weeks of age. The tumor volume in the distal epiphysis of the left and right forelimbs was measured using PTA-enhanced microCT. C, Descriptive statistics of comparison of the tumor volume between males and females. Mean and standard deviation, quartiles, and P-value between groups by Mann-Whitney U test are shown. D, Descriptive statistics of comparison of the tumor volume between left and right arms. Mean and standard deviation, quartiles, and P-value between groups by Mann-Whitney U test are shown.

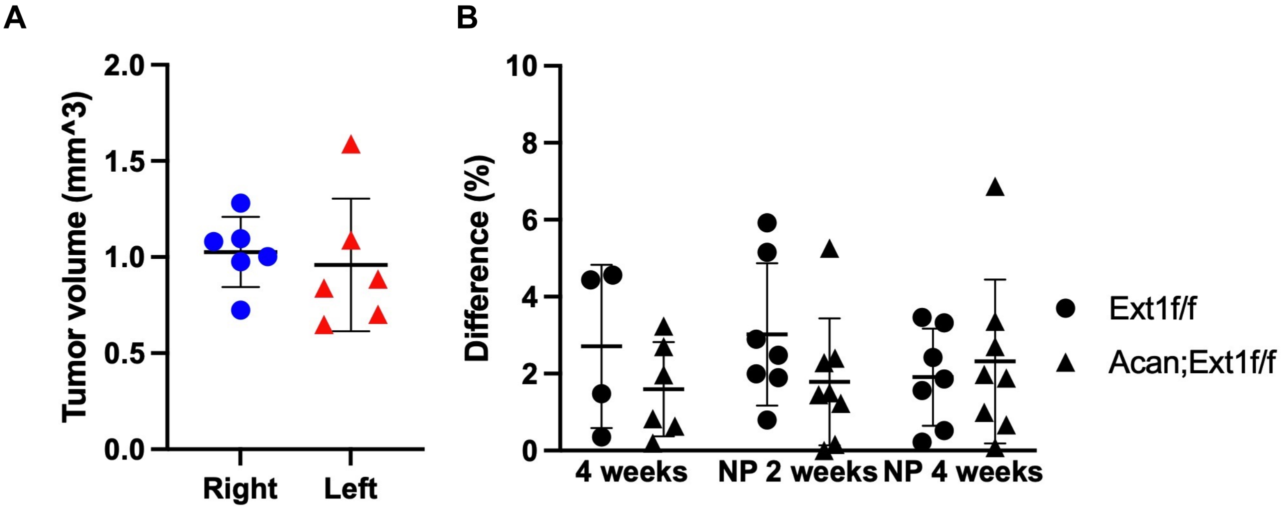

C

	Right	Left
Number of values	6	6
25% Percentile	0.913	0.689
Median	1.04	0.863
75% Percentile	1.14	1.21
IQR	0.227	0.521
Mean	1.03	0.959
Std. Deviation	0.183	0.345
P value	0.39	

D

	Ext1f/f			*AcanCreER;Ext1f/f*		
	4 weeks	NP 2 weeks	NP 4 weeks	4 weeks	NP 2 weeks	NP 4 weeks
Number of values	4	7	7	6	8	8
25% Percentile	0.64	1.9	0.52	0.53	0.43	0.76
Median	2.96	2.48	1.87	1.4	1.48	1.93
75% Percentile	4.53	5.15	3.32	2.84	2.37	3.2
IQR	3.89	3.25	2.8	2.31	1.94	2.45
Mean	2.71	3.02	1.91	1.6	1.79	2.32
Std. Deviation	2.12	1.85	1.26	1.23	1.65	2.13

P value		*Ext1f/f* vs *AcanCreER;Ext1f/f*
	4 weeks	>0.999
	NP 2 weeks	0.31
	NP 4 weeks	>0.999

Supplementary Figure S3. Effects of NRX-NP on tumor growth and bone length. *AcanCreER;Ext1f/f* and *Ext1f/f* mice (n = 6) received tamoxifen injections at P5 and P7, respectively. NRX-204647-loaded (1 μg, 2 μL per limb, Left) or control nanoparticles (2 μL per limb, Right) were injected in the distal epiphysis of the radius and ulna twice a week, starting at 3.5 weeks of age. Some mice were untreated (B, 4 weeks). The forelimbs were harvested at 5.5 weeks (B, NP 2 weeks) or 7.5 weeks of age (A and B, 4 weeks and NP 4 weeks) and 2 or 4 weeks after starting the drug treatment, respectively. A, the tumor volume in the distal epiphysis of the left and right forelimbs was measured using PTA-enhanced microCT. B, The difference in the ulnar bone length between the left and right forelimbs was measured radiographically. Ulnar length was measured from the middle of the distal edge of the primary spongiosa to the center of the trochlear notch of the proximal end. The ratio of the difference to the right ulnar length was calculated. C, Descriptive statistics of comparison of the tumor volume between control NP-injected right and NRX-NP-injected left arms. Mean and standard deviation, quartiles, and P-value between groups by Mann-Whitney U test are shown. D, Descriptive statistics of comparison of the limb length difference between NRX- and control-NP injected limbs. Mean and standard deviation, quartiles, P-value analyzed by Kruskai-Wallis test between groups are shown.

A MA plots

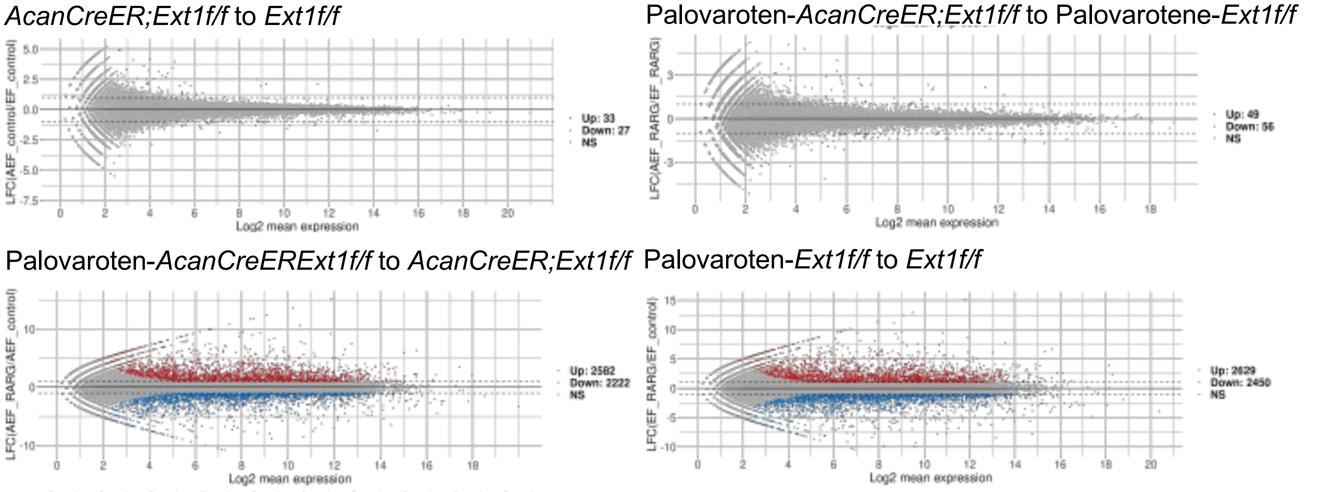

B IPA canonical pathway analysis of the DEGs in palovarotene-treated *Ext1f/f* chondrocytes.

Ingenuity Canonical Pathways	z-score
Axonal Guidance Signaling	NaN
Hepatic Fibrosis / Hepatic Stellate Cell Activation	NaN
Role of Osteoclasts in Rheumatoid Arthritis Signaling Pathway	1.492
Pulmonary Fibrosis Idiopathic Signaling Pathway	1.129
Osteoarthritis Pathway	2.582
GP6 Signaling Pathway	-1.286
Wound Healing Signaling Pathway	0.93
Hepatic Fibrosis Signaling Pathway	3.145
HIF1Œ± Signaling	3.048
S100 Family Signaling Pathway	4.838

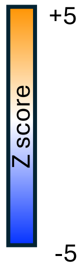

Supplementary Figure S4. RNA-Seq analysis of palovarotene-treated chondrocyte cultures. A, MA plots. Gene expression was analyzed in four sets of comparison groups: *AcanCreER;Ext1f/f* to *Ext1f/f* chondrocyte groups; palovarotene-treated *AcanCreER;Ext1f/f* to untreated *AcanCreER;Ext1f/f* chondrocyte groups; palovarotene-treated *AcanCreER;Ext1f/f* to palovarotene-treated *Ext1f/f* chondrocyte groups; and palovarotene-treated *Ext1f/f* to untreated *Ext1f/f* chondrocyte groups. This set of plots displays the relationship between the expression change (LFC) and average expression (log2 mean expression). Points colored red indicate the upregulated genes with a false discovery rate (FDR) ≤0.05 and an LFC ≥1. Points colored in blue indicate downregulated genes with FDR ≤0.05 and log2-fold-change ≥ ±1. The dashed lines indicate LFC cutoff values of ±1. Gray points indicate genes that were not significantly differentially expressed. B, Top 10 canonical pathways altered in palovarotene-treated *Ext1f/f* chondrocytes compared with that of untreated *Ext1f/f* chondrocytes.

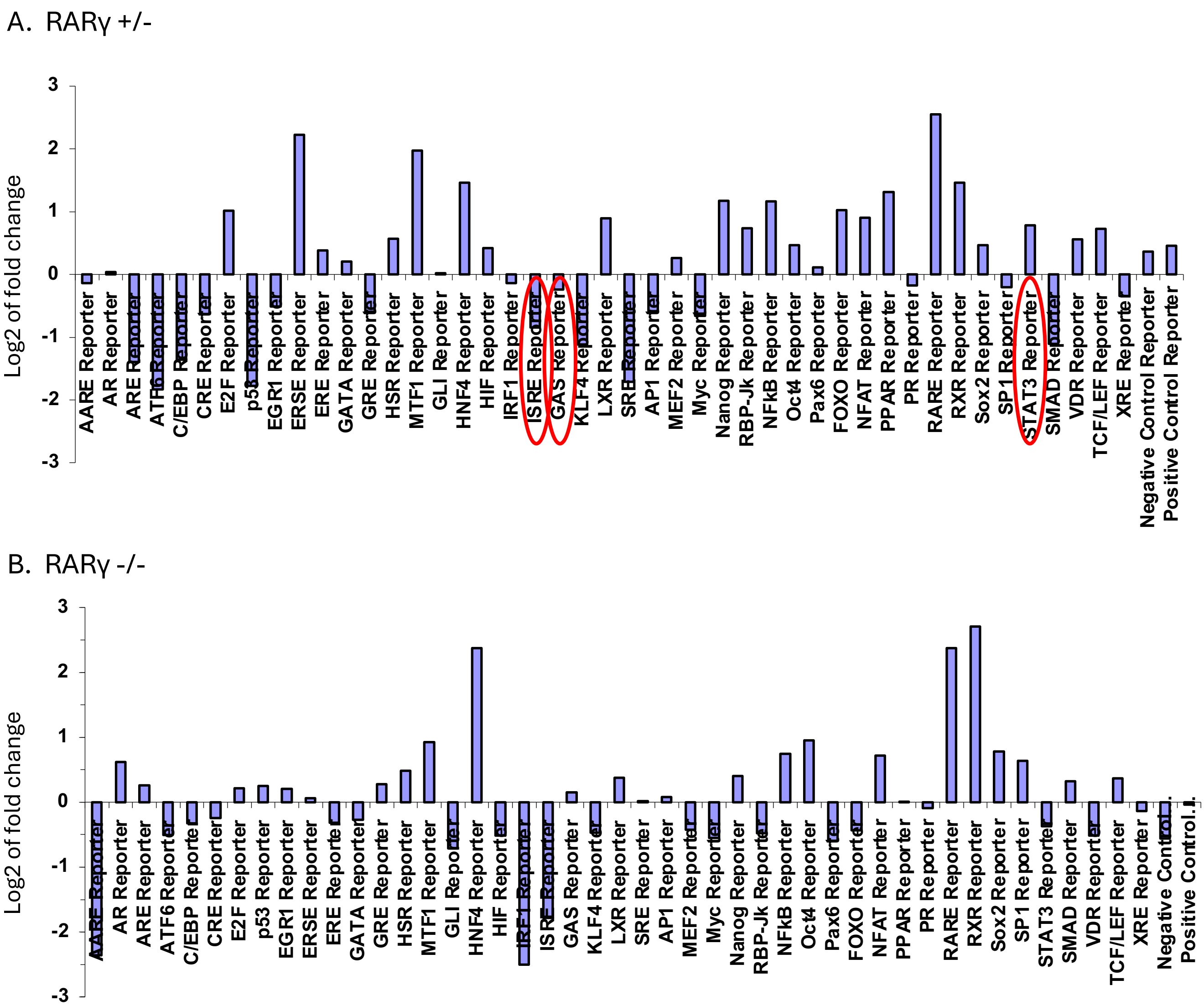

Supplementary Figure S5. Cignal Finder® reporter assay findings. Primary epiphyseal chondrocytes isolated from RARγ hetero (A) or null (B) mice were subjected to reverse transfection with the reporter constructs on the Cignal Finder 45-Pathway Reporter Array plate at a density of 3.0 × 10^4 cells/cm^2. The next day, the cells were treated with 100 nM NRX204647 for 24 h.
